# Predictors of chronic kidney disease in children who underwent surgical correction of congenital heart disease: a long-term follow-up cohort study

**DOI:** 10.1590/2175-8239-JBN-2026-0076en

**Published:** 2026-09-14

**Authors:** Leonor V. Gotuzzo Mendoza, Antonio Cesar Paulillo de Cillo, Fernando Antonialli, Luciana Bertoldi Nucci, Elisa Teixeira Mendes

**Affiliations:** 1 Pontifícia Universidade Católica de Campinas, Escola de Ciências da Vida, Programa de Pós-Graduação Stricto Sensu em Ciências da Saúde, Departamento de Nefrologia Pediátrica, Campinas, SP, Brazil.; 2 Pontifícia Universidade Católica de Campinas, Faculdade de Medicina, Serviço de Nefrologia Pediátrica, Campinas, SP, Brazil.; 3 Pontifícia Universidade Católica de Campinas, Faculdade de Medicina, Serviço de Cirurgia Cardíaca Pediátrica, Campinas, SP, Brazil.; 4 Pontifícia Universidade Católica de Campinas, Escola de Ciências da Vida, Programa de Pós-Graduação Stricto Sensu em Ciências da Saúde, Campinas, SP, Brazil.

**Keywords:** Heart Defects, Congenital, Renal Insufficiency, Chronic, Albuminuria, Pediatrics, Thoracic Surgery, Genetic Syndromes, Kidney Function Tests

## Abstract

**Introduction:**

Chronic kidney disease (CKD) has emerged as a relevant late complication in patients who have undergone surgical correction of congenital heart disease (CHD). Albuminuria has been highlighted as an early marker of subclinical renal injury, which may precede a reduction in glomerular filtration rate (GFR).

**Objective:**

To assess late renal outcomes in patients who underwent surgical correction of CHD and to identify independent predictors for the development of CKD.

**Methods:**

A retrospective cohort study including patients who underwent pediatric cardiac surgery with long-term outpatient follow-up. Renal function was assessed through estimated GFR and the presence of albuminuria. Clinical, surgical, and genetic variables were analyzed using a multivariate logistic regression model.

**Results:**

CKD was observed in 20.6% of patients, while albuminuria was present in 16.4%. In the multivariate analysis, the presence of a genetic syndrome was identified as an independent predictor of CKD (OR 4.06; 95% CI 1.29–12.74). A RACHS-1 surgical score of 2 had a protective effect on renal outcomes.

**Conclusions:**

Genetic syndromes represent a significant risk factor for the progression of renal injury, while surgeries of lower complexity are associated with a better prognosis. These findings reinforce the importance of early screening for albuminuria and long-term nephrological follow-up in this population.

## INTRODUCTION

Acute kidney injury (AKI) after surgery in children with congenital heart disease (CHD) is associated with increased morbidity and worse outcomes in both the immediate and late postoperative periods^1^. With the increasing complexity of procedures, along with improvements in postoperative intensive care, the incidence of AKI in this population has risen from 60 to 500 episodes per 100,000 population/year over the last decade^1^, and it reaches 90% in patients undergoing extracorporeal membrane oxygenation (ECMO)^2^.

In the long term, progression to chronic kidney disease (CKD) may occur even after complete reversal of AKI^3,4^. Numerous mechanisms may be involved, including tissue hypoxia, renal ischemia-reperfusion, inflammatory factors, abnormal fibroblast response, glomerular hypertrophy, inflammatory cell infiltration, and CD4 and IL-1 infiltration, among others^5,6,7,8^. In an experimental ischemia-reperfusion model, Basile et al.^8^ demonstrated the inability to concentrate urine, tubulointerstitial fibrosis, and decreased peritubular capillary density even after 40 weeks following AKI. Studies indicate that patients who develop AKI during hospitalization have up to a 6.7-fold higher risk of progressing to CKD compared to those who do not^9,10,11,12,13^.

Regarding CKD, Greenberg et al.^14^ followed 131 children for a mean follow-up of 5.4 years after heart surgery and identified that 13% had a GFR < 60 mL/min/m^2^, which was not associated with the presence of AKI in the postoperative period. Askenazi et al.^15^ found a 5% progression to terminal CKD in children 3 to 5 years after AKI. Microalbuminuria has been used as an early marker of CKD, with its prevalence in patients with congenital heart disease varying from 18% to 32% in a meta-analysis^16^.

Considering that early diagnosis and management of albuminuria are crucial to prevent the progression of CKD, this study aimed to determine the prevalence of albuminuria in patients with congenital heart disease in the late postoperative period and to identify the main associated risk factors.

## METHODS

This observational study assessed factors associated with late renal injury in children who underwent heart surgery at PUC-Campinas Hospital between 2008 and 2023. The assessment was performed during outpatient follow-up, at least two years after the intervention.

The study was conducted in a university hospital with 325 beds, 29 of which were dedicated to the neonatal and pediatric ICU, along with a referral emergency department and pediatric cardiology outpatient clinic. The hospital is a referral center for the *Sistema Único de Saúde* (SUS, Brazilian Unified Health System) and for private health insurance plans in the areas of pediatric cardiac surgery and pediatric nephrology, with an average of 68 surgeries performed annually.

All children under 13 years of age at the time of surgery who remained in outpatient follow-up at the SUS pediatric cardiology outpatient clinic during the study period (2020–2025) were included.

**Figure 1:**
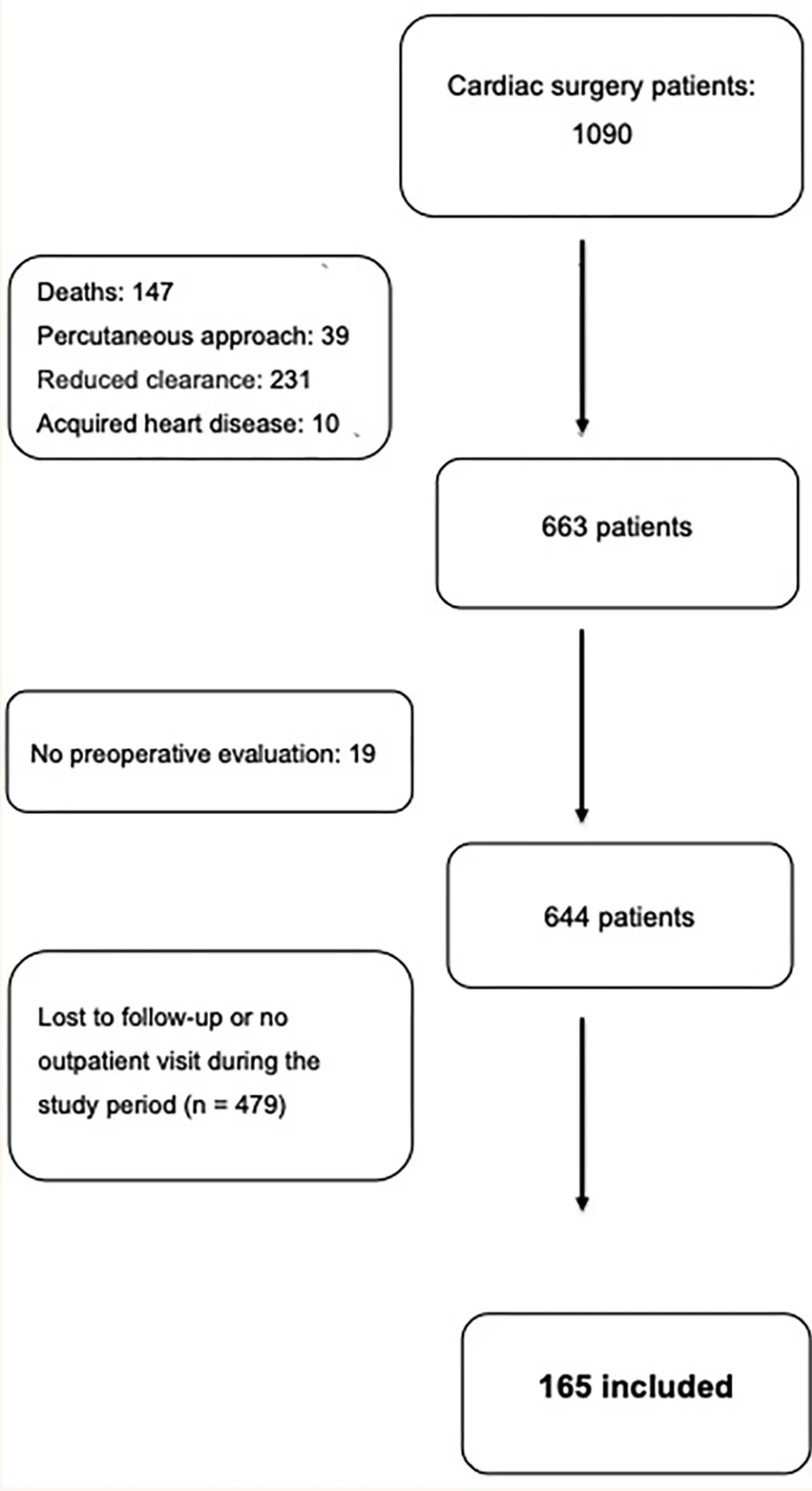
Data collection flowchart.

Patients who did not attend their follow-up appointment during the evaluation period, those with a pre-surgical diagnosis of CKD, patients who underwent catheterization procedures, and those with non-congenital heart diseases were excluded (Figure 1).

Clinical, epidemiological, and cardiac surgery-related data were collected at the time of surgical admission, including sex, age, medical history and comorbidities, type of heart disease, cardiopulmonary bypass (CPB) and aortic cross-clamp duration, use of nephrotoxic drugs, occurrence of AKI, need for dialysis, and presence of sepsis. AKI was classified according to the KDIGO (Kidney Disease: Improving Global Outcomes) criteria.

Surgical complexity was assessed using the RACHS-1^17^ (Risk Adjustment for Congenital Heart Surgery) score, a validated classification system that categorizes congenital heart surgery procedures into six levels according to operative risk, with higher categories indicating greater surgical complexity. During late outpatient follow-up (at least 2 years after surgery), patients were evaluated both clinically and through laboratory evaluation. At this time, non-invasive blood pressure was measured at least three times using a sphygmomanometer and stethoscope, following the auscultatory method. Anthropometric measurements (weight and height) were taken for body mass index (BMI) calculation, based on the World Health Organization curves. Renal function tests, microalbuminuria (albumin/creatinine ratio in a spot urine sample), and estimated creatinine clearance using the Schwartz formula^18^ were assessed.

Statistical analyses were performed using SAS Studio version 3.8. Sociodemographic, epidemiological, and clinical variables were compared according to the clinical outcome (with and without CKD, and with and without AKI). Categorical variables were presented as proportions (%) and compared using the chi-square or Fisher’s exact test. Continuous variables were presented as medians and interquartile ranges (1st [Q1] and 3rd [Q3] quartiles) and compared using the Mann–Whitney test. The level of statistical significance considered was 5% (p < 0.05). After univariate analysis, a multiple logistic regression model was adjusted with variables showing p < 0.20, and the final model retained those with p < 0.05.

## RESULTS

A total of 165 children who underwent cardiac surgery between 2008 and 2023 and returned for follow-up at least two years after the procedure were included in the study. The epidemiological and clinical characteristics of the patients are described in Table 1. The median (Q1–Q3) age at the time of surgery was 0.8 (0.3–2.3) years, and the median (Q1–Q3) follow-up time until the outpatient reassessment was 3.7 (2.7–5.9) years. Most congenital heart defects were non-cyanotic (56.4%), with corrections of atrial septal defect (ASD) and/or ventricular septal defect (VSD) being the most common procedures (35.1%). Regarding disease severity, most cases were classified as RACHS-1 score 2. A diagnosis of genetic syndrome was identified in 33 (20.0%) patients, with Down syndrome being the most prevalent (72.7%). Comorbidities were present in 19 (11.5%) children, and 15.2% had a history of prematurity.

**Table 1:** Clinical, epidemiological, and cardiological characteristics of pediatric patients with and without chronic kidney disease (CKD) who underwent cardiac surgery at PUC-Campinas Hospital (n = 165) between 2008 and 2023.

	Total (n=165)	With CKD (n = 34)	Without CKD (n = 131)	p-value
	n (%) or median (IQR)	n (%) or median (IQR)		
Sex (F:M)	79:86(47.9:52.1)	18:16(52.9:47.1)	61:70(52.4:47.1)	0.5072^a^
Age at the time of surgery (years)	0.8 (0.3–2.3)	0.5 (0.1–1.3)	0.8 (0.3–2.5)	0.0499^b^
Interval between surgery and follow-up (years)	3.7 (2.7–5.9)	3.4 (2.5–5.1)	3.8 (2.7–6.2)	0.3285^b^
Type of heart disease				
Cyanotic	72 (43.6)	16 (47.1)	56 (42.8)	0.6516^a^
Acyanotic	93 (56.4)	18 (52.9)	75 (57.2)	
RACHS-1				
Score of 1	12 (7.3)	3 (8.8)	9 (6.9)	
Score of 2	72 (43.6)	15 (44.1)	57 (43.5)	
Score of 3	64 (38.8)	10 (29.4)	54 (41.2)	0.1911^c^
Score of 4	12 (7.3)	3 (8.8)	9 (6.9)	
Score of 5	0 (0.0)	0 (0.0)	0 (0.0)	
Score of 6	5 (3.0)	3 (8.8)	2 (1.5)	
**Genetic syndromes**	38 (19.9)	12 (35.3)	26 (19.9)	
Down syndrome	26 (70.3)	8 (72.3)	18 (69.2)	0.0566^a^	
DiGeorge syndrome	7 (18.9)	3 (27.3)	4 (15.4)
Williams syndrome	3 (8.1)	0 (0.0)	3 (11.5)	
Comorbidities	25 (15.2)	6 (17.7)	19 (14.5)	
Hypothyroidism	12 (48.0)	1 (16.7)	11 (57.9)	0.6488^a^
Others^e^	13 (52.0)	5 (83.3)	8 (42.1)	
Prematurity	25 (15.2)	3 (8.8)	22 (16.8)	0.2481^a^

Abbreviations – n: number of patients; IQR: interquartile range (Q1–Q3); CKD: chronic kidney disease.

Note – ^a^Chi-square test;

^b^Mann–Whitney U test;

^c^Fisher’s exact test;

^e^Other comorbidities: asthma (n = 2), neurodevelopmental delay (n = 2), cerebral palsy (n = 2), imperforate anus (n = 2), epilepsy (n = 1), cleft palate (n = 1), hydrocephalus (n = 1), lower limb malformations (n = 1).

Variables related to the surgical procedure are presented in Table 2. Most patients (78.2%) underwent CPB, with a median duration of 132 minutes and an aortic cross-clamp duration of 96.5 minutes. Vasoactive drugs were used in 83.6% of cases, and among these, 29.5% of the patients required them for at least 7 days. Nephrotoxic drug use was recorded in 43.0% of patients, with vancomycin being the most commonly used. Acute kidney injury was diagnosed in 49 patients (29.7%) during the postoperative period, of whom 20 were classified as KDIGO stage 3, and 19 required dialysis (11.6%). Sepsis was recorded in 19.5% of patients, and 67.9% required red blood cell transfusion. Mechanical ventilation was necessary in 67.9% of children, with a median duration of 5 days (IQR 2–10). No statistically significant differences were found in these surgical characteristics between patients with and without CKD.

**Table 2:** Surgical characteristics and postoperative outcomes of pediatric patients with and without chronic kidney disease (CKD) who underwent cardiac surgery at PUC-Campinas Hospital between 2008 and 2023.

	Total (n = 165)	With CKD (n = 34)	Without CKD (n = 131)	p-value
	n (%) or median (IQR)	n (%) or median (IQR)	
Underwent CPB	129 (78.2)	26 (76.5)	103 (78.6)	0.7863^a^
CPB duration (minutes)	132 (92–190)	132 (92–205)	132 (92–190)	0.4611^b^
Aortic cross-clamp duration (minutes)	96.5 (57–135)	123 (80–154)	90 (52–132)	0.0863^b^
ECMO during hospitalization	1 (0.6)	0 (0.0)	1 (0.8)	1.0000^c^
Hypothermia*	22 (13.4)	5 (14.7)	17 (13.1)	0.7814^c^
Use of vasoactive drugs	138 (83.6)	31 (91.2)	107 (81.7)	0.1823^a^
Use of vasoactive drugs for > 7 days	41 (29.5)	11 (35.5)	30 (27.8)	0.4069^a^
Acute kidney injury	49 (29.7)	11 (32.4)	38 (29.0)	0.7037^a^
KDIGO					
1	23 (46.9)	3 (27.3)	20 (52.6)	0.2457^c^
2	6 (12.2)	1 (9.1)	5 (13.2)	
3	20 (40.8)	7 (63.6)	13 (34.2)	
Dialysis	19 (11.6)	6 (17.7)	13 (10.0)	0.2326^c^
Use of nephrotoxic drugs	71 (43.0)	19 (55.9)	52 (39.7)	0.0894^a^
Sepsis	32 (19.5)	7 (20.6)	25 (19.2)	0.8589^a^
Red blood cell concentrate	112 (67.9)	22 (64.7)	90 (68.7)	0.6566^a^
Mechanical ventilation	112 (67.9)	25 (73.5)	87 (66.4)	0.4284^a^
Duration of mechanical ventilation (days) (n = 112)	5 (2–10)	7 (2–11)	4 (2–9)	0.0374^b^

Abbreviations – CPB: cardiopulmonary bypass; ECMO: extracorporeal membrane oxygenation; NSAID: non-steroidal anti-inflammatory drug.

Notes – *1 missing value;

anti-inflammatory drug;

^a^Chi-square test;

^b^Mann–Whitney U test;

^c^Fisher’s exact test.

More than one-third of patients were receiving angiotensin-converting enzyme (ACE) inhibitors and/or angiotensin receptor blockers (ARBs) both preoperatively and at late outpatient follow-up (Table 3). All patients had blood pressure within normal limits, and 98.2% had preserved cardiac function. We observed that 34 (20.0%) children had a creatinine clearance below the expected value for their age in the preoperative period.

**Table 3:** Pre- and postoperative renal profile of pediatric patients with and without chronic kidney disease (CKD) who underwent cardiac surgery at PUC-Campinas Hospital between 2008 and 2023.

	Total (n = 165)	With CKD (n = 34)	Without CKD (n = 131)	p-value
	n (%) or median (IQR)	n (%) or median (IQR)	
Preoperative creatinine clearance	104.6 (80.4–144.0)	92.4 (70.9–108.8)	110.9 (83.6–146.7)	0.0112^b^
Postoperative creatinine clearance	138.1 (115.6–156.9)	109.2 (85.2–156.3)	140.3 (123.4–158.6)	0.0157^b^
Preoperative ACEi/ARB use	62 (37.8)	14 (41.2)	48 (36.9)	0.6488^a^
Postoperative ACEi/ARB use	71 (43.0)	15 (44.1)	56 (42.8)	0.8857^a^
Preoperative diuretic use	93 (56.7)	23 (67.7)	70 (53.9)	0.1482^a^
Postoperative diuretic use	68 (41.2)	20 (58.8)	48 (36.6)	0.0192^a^
Microalbuminuria > 30 mg/g creatinine^d^	27 (16.4)	27 (79.4)	0 (0.0)	< 0.0001^a^
Postoperative ejection fraction < 53%	12 (7.8)	3 (10.3)	9 (7.2)	0.6991^c^

Abbreviations – ACEi: angiotensin-converting enzyme inhibitors; ARB: angiotensin receptor blockers.

Notes – ^a^Chi-square test;

^b^Mann–Whitney U test;

^c^Fisher’s exact test;

^d^Interval between surgery and follow-up for microalbuminuria assessment: median 3.7 years (range 2.7–5.9 years).

When assessing urinary sediment as a marker of renal injury, only four patients (2.4%) presented with hematuria, two (1.2%) with leukocyturia, and none showed the presence of red blood cell or leukocyte casts. At late outpatient evaluation, 34 children (20.6%) had CKD, and 27 (16.4%) presented with albuminuria.

In the multivariate analysis (Table 4) the presence of a genetic syndrome remained an independent factor associated with the development of CKD (OR 4.06; 95% CI 1.29–12.74; p = 0.0165). Furthermore, a RACHS-1 severity classification score of 2 was identified as a protective factor. The other variables tested—including age, CPB and aortic cross-clamp duration, use of vasoactive and nephrotoxic drugs, development of AKI in the postoperative period, mechanical ventilation, and sepsis—did not show statistical significance after adjustment.

**Table 4:** Univariate and multivariate logistic regression analysis of factors associated with the development of long-term chronic kidney disease in pediatric patients who underwent cardiac surgery.

	OR (95% CI)	p-value	OR adjusted (95% CI)*	p-value
Age at the time of surgery (years)	0.81 (0.65–1.01)	0.0555	0.98 (0.79–1.26)	0.8912
RACHS-1					
Score of 1	Ref.		Ref.	
Score of 2	0.79 (0.17–5.10)	0.2951	0.024 (0.00–1.28)	0.0517
Score of 3	0.56 (0.11–3.77)		**0.008 (0.00–0.51)**	
Score of 4	1.00 (0.10–9.64)		0.035 (0.00–2.81)	
Score of 6	4.07 (0.31–73.21)		0.084 (0.00–8.10)	
Genetic syndrome (ref. = No)	2.20 (0.97–5.02)	0.0603	4.06 (1.29–12.74)	**0.0165**
Aortic cross-clamp duration (minutes)	1.01 (1.00–1.02)	0.1092	1.01 (1.00–1.02)	0.1168
Use of vasoactive drugs (ref. = No)	2.32 (0.65–8.21)	0.1928	3.04 (0.18–52.04)	0.4436
Use of nephrotoxic drugs (ref. = No)	1.92 (0.90–4.12)	0.0923	0.83 (0.24–2.80)	0.7595
Duration of mechanical ventilation (days)	1.04 (1.00–1.09)	0.0474	1.04 (0.99–1.10)	0.1413

Abbreviations – Ref: reference category; CPB: cardiopulmonary bypass.

Notes – *Adjusted for all variables in the table; **Adjusted using the Firth method.

## DISCUSSION

This study evaluated the impact of surgically corrected congenital heart disease on long-term renal outcomes in pediatric patients.

We identified CKD in 20.6% of patients and albuminuria in 16.4% during late follow-up. The presence of a genetic syndrome was an independent predictor of CKD, whereas a RACHS-1 score of 2 was associated with a lower risk of developing CKD. The meta-analysis by van den Eynde et al.^19^ also associated genetic syndromes with late renal dysfunction in patients with congenital heart disease. These patients are generally more complex due to multifactorial conditions, including their underlying cardiovascular disease. They may also present with other commonly associated comorbidities, such as prematurity. All patients with Down syndrome in this study had atrioventricular septal defects (ASDs + VSDs, complete AVSD, or VSD). These high prevalence rates have also been reported by Benhaourech et al.^20^ (70.4% and 85.2%, respectively).

In this study, AKI was observed in 29.7% of patients, with 11.6% requiring dialysis. In the literature, the occurrence of postoperative AKI varies (3–52%), with a similar minority requiring dialysis (2–9%)^4,5,21,22^. None of the patients who required dialysis showed poor long-term renal outcomes, which has also been reported in other studies that do not associate AKI with worse long-term renal prognosis^23,24^.

In this cohort, renal function assessment may have been influenced by fluid overload, particularly because the estimates were creatinine-based. A persistently positive fluid balance may lead to hemodilution of serum creatinine, potentially masking the occurrence of acute kidney injury^24^. However, daily fluid balance data were not uniformly recorded during the study period, limiting the assessment of their impact on creatinine-based estimations.

Cardiopulmonary bypass was used in 78.2% of procedures, with a mean duration of 132 minutes. In this study, we did not find a relationship between CPB duration and long-term progression to CKD; however, we observed an association between longer CPB duration and the development of AKI during hospitalization (189 minutes vs. 121 minutes). Studies by Sethi et al.^22^ and Li et al.^5^ have also reported a relationship between CPB duration and AKI in children.

Other identified risk factors for AKI were cyanotic heart disease, prematurity, mechanical ventilation, blood transfusion, and sepsis. All of these are considered markers of disease severity and have been described in the literature, although they are not associated with long-term CKD progression in multivariate analyses^4,5,23,24^.

The inclusion of non-cyanotic congenital heart diseases reduced the overall clinical severity of our cohort, with ASD and VSD (35%) accounting for more than one-third of cases. Nevertheless, having cyanotic heart disease was not a predictor of long-term CKD.

In our study, a RACHS-1 score of 2 showed a protective effect for CKD (p = 0.037) in multivariate analysis, indicating a lower risk of adverse renal outcomes among patients undergoing less complex surgical procedures. Higher RACHS-1 scores have been associated with greater long-term renal impairment in other studies, as demonstrated by van den Eynde et al.^19^.

This study showed that 37.8% of patients were receiving ACE inhibitors and/or ARBs before surgery and 43% at the late outpatient evaluation. The use of these medications was not associated with progression to CKD. These drugs were initially used for diabetic nephropathy and are considered nephroprotective for that population. Their use is well established for controlling microalbuminuria and consequently for renal protection, including in the pediatric population^25,26^.

Albuminuria is an early marker of renal injury in congenital heart disease, even in the presence of preserved GFR. We found microalbuminuria in 16.4% of patients in our late follow-up analysis. A meta-analysis including 873 individuals after Fontan surgery identified albuminuria in 28.4% of patients, associated with mechanisms of venous congestion and endothelial dysfunction, suggesting that renal impairment may occur subclinically and precede the decline in excretory function^16^. In 2020, a study involving 58 neonates found 17% with CKD, 12% with microalbuminuria, and 12% with hypertension, with postoperative cyanosis being the only independent predictor for CKD^27^.

This study has some limitations. First, its retrospective design restricted the analysis to patients who survived and maintained long-term outpatient follow-up. Second, the inclusion of acyanotic congenital heart disease contributed to clinical heterogeneity. In addition, acute kidney injury may have been underestimated due to the lack of fluid balance data, and microalbuminuria was assessed at a single time point. Although no prior sample size calculation was performed, a *post hoc* analysis confirmed adequate statistical power for the main comparisons.

On the other hand, this is the largest Brazilian cohort study evaluating renal prognosis in children with congenital heart disease who underwent surgical procedures.

In conclusion, our findings reinforce that microalbuminuria should be incorporated into the follow-up of patients with congenital heart disease as an early marker of progressive renal dysfunction, especially in those with more complex congenital heart disease (higher RACHS-1 scores) and those with genetic syndromes.

## Data Availability

The datasets generated during this study are available from the corresponding author on reasonable request.
