# Supplementary material for: Predictors of chronic kidney disease in children who underwent surgical correction of congenital heart disease: a long-term follow-up cohort study
Source: J Bras Nefrol. 2026 Sep 14;48(4):e20260076. doi: 10.1590/2175-8239-JBN-2026-0076en (PMC13595991; doi:10.1590/2175-8239-JBN-2026-0076en)
Supplement: Translation

# Translation: Preditores de disfunção renal crônica em crianças submetidas à cirurgia para correção de cardiopatias congênitas em seguimento tardio: estudo de coorte

**DOI:** 10.1590/2175-8239-JBN-2026-0076pt

**Authors:** Leonor V. Gotuzzo Mendoza, Antonio Cesar Paulillo de Cillo, Fernando Antonialli, Luciana Bertoldi Nucci, Elisa Teixeira Mendes

**Affiliations:** 1 Pontifícia Universidade Católica de Campinas, Escola de Ciências da Vida, Programa de Pós-Graduação Stricto Sensu em Ciências da Saúde, Departamento de Nefrologia Pediátrica, Campinas, SP, Brasil.; 2 Pontifícia Universidade Católica de Campinas, Faculdade de Medicina, Serviço de Nefrologia Pediátrica, Campinas, SP, Brasil.; 3 Pontifícia Universidade Católica de Campinas, Faculdade de Medicina, Serviço de Cirurgia Cardíaca Pediátrica, Campinas, SP, Brasil.; 4 Pontifícia Universidade Católica de Campinas, Escola de Ciências da Vida, Programa de Pós-Graduação Stricto Sensu em Ciências da Saúde, Campinas, SP, Brasil.

**Keywords:** Cardiopatias Congênitas, Insuficiência Renal Crônica, Albuminúria, Pediatria, Cirurgia Torácica, Síndromes Genéticas, Testes de Função Renal.

## Abstract

**Introdução:**

A doença renal crônica (DRC) é uma complicação tardia relevante em pacientes submetidos à correção cirúrgica de cardiopatias congênitas (CC). A albuminúria vem sendo reconhecida como marcador precoce de lesão renal subclínica, podendo anteceder a redução da taxa de filtração glomerular (TFG).

**Objetivo:**

Avaliar a função renal em seguimento tardio de pacientes submetidos à correção cirúrgica de CC e identificar preditores independentes de DRC.

**Métodos:**

Estudo de coorte retrospectivo que incluiu pacientes submetidos à cirurgia cardíaca pediátrica com seguimento ambulatorial de longo prazo. A função renal foi avaliada pela TFG estimada e pela presença de albuminúria. Variáveis clínicas, cirúrgicas e genéticas foram analisadas por regressão logística multivariada.

**Resultados:**

A DRC foi identificada em 20,6% dos pacientes e a albuminúria em 16,4%. Na análise multivariada, a presença de síndrome genética associou-se significativamente ao desenvolvimento de DRC (OR 4,06; IC95% 1,29–12,74). O escore cirúrgico RACHS-1 igual a 2 apresentou efeito protetor sobre os desfechos renais.

**Conclusões:**

Síndromes genéticas constituem um importante fator de risco para a progressão da lesão renal, enquanto cirurgias de menor complexidade associam-se a melhor prognóstico. Os achados reforçam a importância da triagem precoce por albuminúria e do acompanhamento nefrológico em longo prazo nessa população.

## INTRODUÇÃO

A lesão renal aguda (LRA) após cirurgia em crianças com cardiopatia congênita (CC) está associada a maior morbidade e piores desfechos tanto no período pós-operatório imediato quanto no tardio^1^. Devido ao aumento da complexidade dos procedimentos cirúrgicos, associado aos avanços no cuidado intensivo pós-operatório, a incidência de LRA nessa população tem aumentado na última década, variando de 60 a 500 casos por 100.000 habitantes/ano^1^, podendo chegar a até 90% entre pacientes submetidos à oxigenação por membrana extracorpórea (ECMO)^2^.

A progressão para doença renal crônica (DRC), contudo, pode ocorrer a longo prazo, mesmo com a recuperação da função renal no período perioperatório^3,4^. Diversos mecanismos parecem estar envolvidos nessa evolução tardia, tais como hipóxia tecidual, lesão de isquemia-reperfusão, ativação inflamatória, resposta fibroblástica anormal, hipertrofia glomerular e infiltração de células inflamatórias, incluindo CD4 e IL-1^5,6,7,8^. Em um modelo experimental, Basile et al.^8^ demonstraram alterações persistentes, como incapacidade de concentrar urina, fibrose túbulo-intersticial e redução da densidade capilar peritubular, mesmo após 40 semanas do episódio de LRA. Estudos clínicos indicam que pacientes que desenvolvem LRA durante a internação apresentam risco até 6,7 vezes maior de evoluir para DRC quando comparados àqueles sem LRA^9,10,11,12,13^.

Greenberg et al.^14^ acompanharam 131 crianças por um período médio de 5,4 anos após cirurgia cardíaca e observaram que 13% apresentavam TFG < 60 mL/min/m^2^, sem associação com LRA no pós-operatório. Já Askenazi et al.^15^ identificaram progressão para DRC terminal em 5% dos casos entre 3 e 5 anos após a ocorrência de LRA. A microalbuminúria tem sido utilizada como marcador precoce de lesão renal e apresenta prevalência variando de 18% a 32% em pacientes com cardiopatias congênitas, conforme meta-análise^16^.

Diante disso, devido à importância do diagnóstico precoce da DRC, este estudo teve como objetivo determinar a prevalência de albuminúria em pacientes com cardiopatia congênita no seguimento tardio pós-operatório, bem como identificar os principais fatores de risco associados à evolução para DRC a longo prazo.

## MÉTODOS

Trata-se de um estudo observacional que avaliou fatores associados à lesão renal tardia em crianças submetidas à cirurgia cardíaca no Hospital da PUC-Campinas entre 2008 e 2023. A avaliação foi realizada durante o seguimento ambulatorial, no mínimo dois anos após o procedimento cirúrgico.

O estudo ocorreu em um hospital universitário com 325 leitos, sendo 29 destinados à UTI neonatal e pediátrica, além de pronto atendimento de referência e ambulatório de cardiologia pediátrica. A instituição é centro de referência tanto para o Sistema Único de Saúde (SUS) quanto para a saúde suplementar nas áreas de cirurgia cardíaca pediátrica e nefrologia pediátrica, com média anual de 68 cirurgias.

Foram incluídas crianças menores de 13 anos no momento cirúrgico, que compareceram ao seguimento ambulatorial no serviço de cardiologia pediátrica do SUS durante o período do estudo (2020–2025). Excluíram-se pacientes faltantes à consulta de reavaliação, aqueles com diagnóstico prévio de doença renal crônica (DRC), submetidos a procedimentos de cateterismo (não cirúrgicos) e aqueles com cardiopatias não congênitas (Figura 1).

**Figura 1:** Fluxograma da coleta de dados.

Foram coletados dados clínicos, epidemiológicos e relacionados ao procedimento cirúrgico no momento da internação, incluindo sexo, idade, comorbidades, tipo de cardiopatia, tempo de circulação extracorpórea (CEC) e de pinçamento aórtico, uso de drogas nefrotóxicas, ocorrência de LRA, necessidade de diálise e presença de sepse. A LRA foi definida conforme os critérios do KDIGO.

A classificação da complexidade do procedimento cirúrgico foi realizada pelo escore RACHS-1 (*Risk Adjustment for Congenital Heart Surgery*)^17^, um escore de classificação validado que categoriza os procedimentos de cirurgia cardíaca congênita em seis níveis, de acordo com o risco operatório, sendo que categorias mais elevadas indicam maior complexidade cirúrgica. No seguimento tardio, os pacientes foram avaliados clinicamente e por exames laboratoriais. A pressão arterial foi aferida pelo método auscultatório em pelo menos três medidas. Também foram realizadas medidas antropométricas para cálculo do índice de massa corporal (IMC), conforme as curvas da Organização Mundial da Saúde. A função renal foi avaliada por meio da TFG estimada (fórmula de *Schwartz*)^18^ e da relação albumina/creatinina em amostra isolada de urina.

A análise estatística foi realizada no software SAS versão 3.8. As variáveis foram relacionadas com os desfechos clínicos (presença ou ausência de DRC e ocorrência de LRA). Variáveis categóricas foram expressas em proporções (%) e comparadas pelos testes *qui-quadrado* ou *Fisher*. Variáveis contínuas foram apresentadas como mediana e intervalo interquartil (Q1–Q3) e comparadas pelo teste de *Mann–Whitney*. Adotou-se um nível de significância de 5% (p < 0,05). Variáveis com p < 0,20 na análise univariada foram incluídas no modelo de regressão logística multivariada, permanecendo no modelo final aquelas com p < 0,05.

## RESULTADOS

Foram incluídas 165 crianças submetidas à cirurgia cardíaca entre 2008 e 2023, que retornaram para acompanhamento ambulatorial pelo menos dois anos após o procedimento. As características epidemiológicas e clínicas dos pacientes estão descritas na Tabela 1. A mediana (Q1–Q3) de idade no momento da cirurgia foi de 0,8 (0,3–2,3) anos, e o tempo mediano (Q1–Q3) de seguimento até a reavaliação ambulatorial foi de 3,7 (2,7–5,9) anos. A maioria das cardiopatias congênitas era acianótica (56,4%), sendo a correção de comunicação interatrial (CIA) e/ou comunicação interventricular (CIV) o procedimento mais frequente (35,1%). Quanto à complexidade cirúrgica, a maioria dos casos foi classificada como escore RACHS-1 igual a 2. O diagnóstico de síndrome genética foi identificado em 33 (20,0%) pacientes, sendo a síndrome de Down a mais prevalente (72,7%). Comorbidades estavam presentes em 19 (11,5%) crianças, e 15,2% tinham história de prematuridade.

**Tabela 1:** Análise univariada de fatores de risco associados à evolução para doença renal crônica (DRC) entre pacientes pediátricos submetidos à cirurgia cardíaca no período de 2008 a 2023 no Hospital PUC-Campinas (n = 165).

	Total (n = 165)	Com DRC (n = 34)	Sem DRC (n = 131)	Valor de P
	n (%) ou mediana (IIQ)	n (%) ou mediana (IIQ)	
Sexo (F:M)	79:86 (47,9:52,1)	18:16 (52,9:47,1)	61:70 (52,4:47,1)	0,5072^a^
Idade no momento da cirurgia (anos)	0,8 (0,3–2,3)	0,5 (0,1–1,3)	0,8 (0,3–2,5)	0,0499^b^
Intervalo entre cirurgia e avaliação ambulatorial (anos)	3,7 (2,7–5,9)	3,4 (2,5–5,1)	3,8 (2,7–6,2)	0,3285^b^
Cardiopatia cianótic^a^	72 (43,6)	16 (47,1)	56 (42,8)	0,6516^a^
Cardiopatia acianótic^a^	93 (56,4)	18 (52,9)	75 (57,2)	
RACHS-1				
Escore 1	12 (7,3)	3 (8,8)	9 (6,9)	
Escore 2	72 (43,6)	15 (44,1)	57 (43,5)	
Escore 3	64 (38,8)	10 (29,4)	54 (41,2)	0,1911^c^
Escore 4	12 (7,3)	3 (8,8)	9 (6,9)	
Escore 5	0 (0,0)	0 (0,0)	0 (0,0)	
Escore 6	5 (3,0)	3 (8,8)	2 (1,5)	
**Síndromes genéticas**	38 (19,9)	12 (35,3)	26 (19,9)	
Síndrome de Down	26 (70,3)	8 (72,3)	18 (69,2)	0,0566^a^
Síndrome DiGeorge	7 (18,9)	3 (27,3)	4 (15,4)	
Síndrome de Williams	3 (8,1)	0 (0,0)	3 (11,5)	
Comorbidades	25 (15,2)	6 (17,7)	19 (14,5)	
Hipotireoidismo	12 (48,0)	1 (16,7)	11 (57,9)	0,6488^a^
Outras^e^	13 (52,0)	5 (83,3)	8 (42,1)	
Prematuridade	25 (15,2)	3 (8,8)	22 (16,8)	0,2481^a^

Abreviaturas – n: número de pacientes; IIQ: intervalo interquartil (Q1–Q3); DRC: doença renal crônica.

Nota – ^a^Teste do qui-quadrado;

^b^Teste de Mann–Whitney;

^c^Teste exato de Fisher;

^e^Outras comorbidades: asma (n = 2), atraso no neurodesenvolvimento (n = 2), paralisia cerebral (n = 2), ânus imperfurado (n = 2), epilepsia (n = 1), fissura palatina (n = 1), hidrocefalia (n = 1) e malformações de membros inferiores (n = 1).

As variáveis relacionadas ao procedimento cirúrgico estão apresentadas na Tabela 2. A maioria dos pacientes (78,2%) foi submetida à circulação extracorpórea (CEC), com tempo mediano de 132 minutos e de pinçamento aórtico de 96,5 minutos. Drogas vasoativas foram utilizadas em 83,6% dos casos, sendo que 29,5% destes as utilizaram por pelo menos 7 dias. Medicamentos nefrotóxicos foram usados em 43,0% dos pacientes, sendo a vancomicina a mais utilizada. A LRA foi diagnosticada em 49 pacientes (29,7%) no período pós-operatório, dos quais 20 foram classificados como KDIGO 3, e 19 necessitaram de diálise (11,6%). Quadro séptico ocorreu em 19,5% dos pacientes, e 67,9% receberam concentrado de hemácias. Ventilação mecânica ocorreu em 67,9% das crianças, com duração mediana de 5 dias (IIQ 2–10).

**Tabela 2:** Características cirúrgicas e desfechos pós-operatórios de pacientes pediátricos com e sem doença renal crônica (DRC) submetidos à cirurgia cardíaca no Hospital PUC-Campinas entre 2008 e 2023.

	Total (n = 165)	Com DRC (n = 34)	Sem DRC (n = 131)	Valor de P
	n (%) ou mediana (IIQ)	n (%) ou mediana (IIQ)	
Submetido à CEC	129 (78,2)	26 (76,5)	103 (78,6)	0,7863^a^
Duração da CEC (minutos)	132 (92–190)	132 (92–205)	132 (92–190)	0,4611^b^
Clampeamento aórtico Duração (minutos)	96,5 (57–135)	123 (80–154)	90 (52–132)	0,0863^b^
ECMO durante internação	1 (0,6)	0 (0,0)	1 (0,8)	1,0000^c^
Hipotermia*	22 (13,4)	5 (14,7)	17 (13,1)	0,7814c
Uso de droga vasoativa	138 (83,6)	31 (91,2)	107 (81,7)	0,1823^a^
> 7 dias de droga vaso ativa	41 (29,5)	11 (35,5)	30 (27,8)	0,4069^a^
Insuficiência renal aguda	49 (29,7)	11 (32,4)	38 (29,0)	0,7037^a^
KDIGO				
1	23 (46,9)	3 (27,3)	20 (52,6)	
2	6 (12,2)	1 (9,1)	5 (13,2)	0,2457c
3	20 (40,8)	7 (63,6)	13 (34,2)	
Diálise	19 (11,6)	6 (17,7)	13 (10,0)	0,2326c
Uso de drogas nefrotóxicas	71 (43,0)	19 (55,9)	52 (39,7)	0,0894^a^
Sepse	32 (19,5)	7 (20,6)	25 (19,2)	0,8589^a^
Transfusão sanguínea	112 (67,9)	22 (64,7)	90 (68,7)	0,6566^a^
Ventilação mecânica	112 (67,9)	25 (73,5)	87 (66,4)	0,4284^a^
Duração da ventilação mecânica (dias) (n = 112)	5 (2–10)	7 (2–11)	4 (2–9)	0,0374^b^

Abreviaturas – CEC: circulação extracorpórea; ECMO: oxigenação por membrana extracorpórea; AINE: anti-inflamatório não esteroidal.

Notas – *1 dado ausente;

^a^Teste do qui-quadrado;

^b^Teste de Mann–Whitney;

^c^Teste exato de Fisher.

Mais de um terço dos pacientes fazia uso de inibidores da enzima conversora de angiotensina (IECA) e/ou bloqueadores dos receptores de angiotensina (BRA) tanto no período pré-operatório quanto no seguimento ambulatorial tardio (Tabela 3). Todos os pacientes apresentavam níveis pressóricos dentro da normalidade, e 98,2% tinham função cardíaca preservada.

**Tabela 3:** Perfil renal pré- e pós-operatório de pacientes pediátricos com e sem doença renal crônica (DRC) submetidos à cirurgia cardíaca entre 2008 e 2023 no Hospital PUC-Campinas.

	Total (n = 165)	Com DRC (n = 34)	Sem DRC (n = 131)	Valor de P
	n (%) ou mediana (IIQ)	n (%) ou mediana (IIQ)	
*Clearance* de creatinina pré-operatória	104,6 (80,4–144,0)	92,4 (70,9–108,8)	110,9 (83,6–146,7)	0,0112^b^
*Clearance* de creatinina pós-operatória	138,1 (115,6–156,9)	109,2 (85,2–156,3)	140,3 (123,4–158,6)	0,0157^b^
IECA/BRA pré-operatório	62 (37,8)	14 (41,2)	48 (36,9)	0,6488^a^
IECA/BRA pós-operatório	71 (43,0)	15 (44,1)	56 (42,8)	0,8857^a^
Diurético pré-operatório	93 (56,7)	23 (67,7)	70 (53,9)	0,1482^a^
Diurético pós-operatório	68 (41,2)	20 (58,8)	48 (36,6)	0,0192^a^
Microalbuminúria > 30 mg/g creatinina^d^	27 (16,4)	27 (79,4)	0 (0,0)	< 0,0001^a^
Fração de ejeção pós-operatória < 53%	12 (7,8)	3 (10,3)	9 (7,2)	0,6991^c^

Abreviaturas – IECA: inibidores da enzima conversora da angiotensina; BRA: bloqueadores dos receptores da angiotensina.

Notas – ^a^Teste do qui-quadrado;

^b^Teste de Mann–Whitney;

^c^Teste exato de Fisher;

^d^Intervalo (anos) entre a cirurgia e o seguimento ambulatorial (avaliação da microalbuminúria) – mediana de 3,7 anos (IIQ: 2,7–5,9).

Em relação à análise do sedimento urinário, apenas quatro pacientes (2,4%) apresentaram hematúria, dois (1,2%) apresentaram leucocitúria e nenhum apresentou cilindros hemáticos e/ou leucocitários. Na avaliação ambulatorial tardia, 34 crianças (20,6%) apresentavam DRC e 27 (16,4%) apresentavam albuminúria.

Na análise multivariada (Tabela 4), a presença de síndrome genética manteve-se como fator independente associado ao desenvolvimento de DRC (OR 4,06; IC 95% 1,29–12,74; p = 0,0165). Adicionalmente, a classificação de gravidade RACHS-1 igual a 2 foi identificada como fator protetor. As demais variáveis analisadas — incluindo idade, tempo de CEC e de pinçamento aórtico, uso de drogas vasoativas e nefrotóxicas, ocorrência de LRA no pós-operatório, ventilação mecânica e sepse — não apresentaram significância estatística após ajuste em análise multivariada.

**Tabela 4:** Análise de regressão logística univariada e multivariada dos fatores associados ao desenvolvimento de doença renal crônica em longo prazo em pacientes pediátricos submetidos à cirurgia cardíaca.

	OR (IC 95%)	Valor de P	OR ajustado (IC 95%)*	Valor de P
Idade no momento da cirurgia (anos)	0,81 (0,65–1,01)	0,0555	0,98 (0,79–1,26)	0,8912
RACHS-1				
Escore 1	Ref.		Ref.	
Escore 2	0,79 (0,17–5,10)	0,2951	0,024 (0,00–1,28)	0,0517
Escore 3	0,56 (0,11–3,77)		**0,008 (0,00–0,51)**	
Escore 4	1,00 (0,10–9,64)		0,035 (0,00–2,81)	
Escore 6	4,07 (0,31–73,21)		0,084 (0,00–8,10)	
Síndrome genética (ref. = não)	2,20 (0,97–5,02)	0,0603	4,06 (1,29–12,74)	**0,0165**
Duração do clampeamento aórtico (minutos)	1,01 (1,00–1,02)	0,1092	1,01 (1,00–1,02)	0,1168
Uso de drogas vasoativas (ref. = Não)	2,32 (0,65–8,21)	0,1928	3,04 (0,18–52,04)	0,4436
Uso de drogas nefrotóxicas (ref. = Não)	1,92 (0,90–4,12)	0,0923	0,83 (0,24–2,80)	0,7595
Duração de ventilação mecânica (dias)	1,04 (1,00–1,09)	0,0474	1,04 (0,99–1,10)	0,1413

Abreviaturas – Ref: categoria de referência; CEC: circulação extracorpórea.

Notas – *Ajustado para todas as variáveis incluídas na tabela; **Ajustado pelo método de Firth.

## DISCUSSÃO

Este estudo avaliou o impacto da cardiopatia congênita com correção cirúrgica sobre os desfechos renais em longo prazo em pacientes pediátricos.

Identificamos doença renal crônica (DRC) em 20,6% dos pacientes e albuminúria em 16,4% no seguimento tardio. A presença de síndrome genética foi um preditor independente de DRC, enquanto um escore RACHS-1 igual a 2 foi um fator protetor para esse desfecho. A meta-análise de van den Eynde et al.^19^ também demonstrou associação entre síndromes genéticas e disfunção renal tardia em pacientes com cardiopatia congênita. Tais pacientes apresentam maior complexidade clínica de forma multifatorial, que inclui a própria doença cardiovascular, além de comorbidades frequentemente associadas, como prematuridade. No presente estudo, todos os pacientes com síndrome de Down apresentaram defeitos do septo atrioventricular (ASD + VSD, AVSD completo ou VSD). Esse padrão também foi descrito por Benhaourech et al.^20^, com prevalências de 70,4% e 85,2%, respectivamente.

A LRA ocorreu em 29,7% dos pacientes, sendo que 11,6% necessitaram de diálise. Na literatura, a incidência de LRA no pós-operatório varia entre 3% e 52%, com uma pequena proporção dos pacientes necessitando de diálise (2–9%)^4,5,21,22^. Neste estudo, pacientes que necessitaram de terapia dialítica não apresentaram pior evolução renal a longo prazo. Diversos estudos também não demonstraram associação entre LRA e pior prognóstico renal tardio^23,24^.

É importante considerar que, nesta coorte, a avaliação da função renal pode ter sido influenciada pela sobrecarga volêmica, especialmente porque as estimativas foram baseadas na dosagem da creatinina. Um balanço hídrico persistentemente positivo pode levar à hemodiluição da creatinina sérica, mascarando a ocorrência de LRA e levando à subestimação de sua gravidade^24^. No entanto, dados de balanço hídrico não estavam disponíveis de forma regular ao longo do período do estudo, limitando a avaliação do impacto desse fator sobre as estimativas de função renal.

A CEC foi utilizada em 78,2% dos procedimentos, com tempo médio de 132 minutos. Não foi observada associação entre o tempo de CEC e a progressão para DRC em longo prazo; entretanto, identificamos relação entre maior duração de CEC e ocorrência de LRA durante a internação (189 minutos vs. 121 minutos). Estudos de Sethi et al.^22^ e Li et al.^5^ também demonstraram associação entre maior duração de CEC e desenvolvimento de LRA em crianças.

Outros fatores de risco identificados para LRA incluíram cardiopatia cianótica, prematuridade, ventilação mecânica, transfusão sanguínea e sepse. Todos esses fatores são reconhecidos como marcadores de maior gravidade clínica e são amplamente descritos na literatura, embora não tenham se mostrado associados à progressão para DRC em longo prazo nas análises multivariadas^4,5,23,24^.

A inclusão de cardiopatias não cianóticas reduziu a gravidade global da coorte, com defeitos do septo atrial e ventricular (ASD e VSD) representando cerca de 35% dos casos. Ainda assim, a presença de cardiopatia cianótica não foi associada a um pior prognóstico renal a longo prazo.

Neste estudo, um escore RACHS-1 igual a 2 apresentou efeito protetor para DRC (p = 0,037) na análise multivariada, indicando que a menor complexidade cirúrgica é um marcador de proteção para DRC em longo prazo. Escores mais elevados de RACHS-1 têm sido associados a maior comprometimento renal tardio na literatura, conforme demonstrado por van den Eynde et al.^19^.

Observamos que 37,8% dos pacientes faziam uso de inibidores da enzima conversora de angiotensina (IECA) e/ou bloqueadores do receptor de angiotensina (BRA) no período pré-operatório, e 43% durante o seguimento ambulatorial tardio. O uso dessas medicações não se associou à progressão para DRC. Esses fármacos foram inicialmente empregados no contexto da nefropatia diabética e hoje são considerados nefroprotetores nessa população. Seu uso está bem estabelecido no controle da microalbuminúria e, consequentemente, na proteção renal, inclusive em pacientes pediátricos^25,26^.

A albuminúria é reconhecida como marcador precoce de lesão renal em pacientes com cardiopatia congênita, mesmo na presença de taxa de filtração glomerular preservada. Foi identificada microalbuminúria em 16,4% dos pacientes na avaliação tardia. Uma meta-análise incluindo 873 indivíduos após cirurgia de Fontan demonstrou prevalência de albuminúria de 28,4%, associada a mecanismos de congestão venosa e disfunção endotelial, sugerindo que o comprometimento renal pode ocorrer de forma subclínica e preceder a redução da função excretora^16^. Em 2020, um estudo envolvendo 58 neonatos descreveu prevalência de 17% de DRC, 12% de microalbuminúria e 12% de hipertensão, sendo a cianose pós-operatória o único preditor independente de DRC^27^.

Este estudo apresenta limitações. Primeiramente, seu delineamento retrospectivo limita a análise aos pacientes que sobreviveram e mantiveram seguimento ambulatorial em longo prazo. Além disso, a inclusão de cardiopatias congênitas acianóticas contribuiu para a redução global da gravidade da amostra. Como já mencionado, a LRA pode ter sido subestimada devido à ausência de dados de balanço hídrico. A microalbuminúria foi avaliada em um único momento, limitando a análise de sua evolução temporal. Embora não tenha sido realizado cálculo prévio do tamanho amostral, a análise *post hoc* demonstrou poder estatístico adequado para as principais comparações.

Apesar das limitações descritas, este representa o maior estudo brasileiro avaliando o prognóstico renal em crianças com cardiopatia congênita submetidas à correção cirúrgica.

Em conclusão, nossos achados reforçam que a microal­buminúria deve ser incorporada ao seguimento de pacientes com cardiopatia congênita como marcador precoce de disfunção renal progressiva, especialmente em indivíduos com maior complexidade cardíaca (escores RACHS-1 mais elevados) e em pacientes com síndromes genéticas.

## Data Availability

Os conjuntos de dados utilizados e/ou analisados durante o presente estudo estão disponíveis mediante solicitação razoável à autora correspondente.
